# Spectrum of EGFR mutation and its relation with high-risk predictors in thyroid cancer in Kashmiri population: 2 years prospective study at a tertiary care hospital

**DOI:** 10.1186/s43046-022-00139-y

**Published:** 2022-10-17

**Authors:** Tariq Ahmad Mir, Ajaz Qadir, Munir Ahmad Wani, Muzafar Maqsood Wani

**Affiliations:** 1 Department of Pediatric Surgery, SKIMS, Jammu and Kashmir, Kitchama, Sheeri, Baramulla, JK 193101 India; 2 Department of Endocrinology, SKIMS, Jammu and Kashmir, Rajpora, District, Pulwama, JK 192306 India; 3 Department of General and Minimal Invasive Surgery, SKIMS, Jammu and Kashmir, Pakherpora, Tehsil, Chare-shareef, 191112 India; 4 Department of Nephrology, SKIMS, Jammu and Kashmir, Wani Manzil, Chandpora, Harwan, JK 191123 India

**Keywords:** Papillary carcinoma (PC), Follicular carcinoma (FC), Epidermal growth factor receptor (EGFR)

## Abstract

**Background:**

EGFR mutation has not been extensively studied in thyroid cancer. This study was conducted to study spectrum of EGFR mutation in thyroid cancer in Kashmiri population for possible therapeutic purpose.

**Methods:**

It was 2 years prospective cross-sectional study conducted at a tertiary care center in which histologically confirmed, untreated thyroid cancers were included. These specimens were subjected to EGFR mutation analysis by AS-PCR method.

**Results:**

There were a total 60 patients with preponderance of females [44(73%) vs 16(27%)]. Most were in the age group of less than 45 years (75%). Most of these patients were non-smokers [50(83.3%) vs 10 (17.3%)]. Papillary thyroid carcinoma (PTC) was the commonest type 48(80%), rest was follicular type (FTC) 12(20%). Well-differentiated carcinoma (WDC) was common than poorly differentiated (PDC) [41(68.4%) vs 19 (31.6%)]. Lymph node metastasis and vascular invasion were present in 32 (53.4%) and 17 (28.4%) respectively. Thirty-two (53.3%) patients were having 15 bp deletion in exon 19 of EGFR*.* These deletions were common in PTC than FTC, 29(60.5%) vs 3(25%) which was statistically significant (*p* = 0.04, CI = 0.2). The total mutational rate of T790M in EGFR tyrosine kinase domain (exon 20) was found to be only 8.4% (5 of 60). Only 4 (8.3%) of these mutations were detected in PTC and rest in FTC (1 of 12). Twenty-six (43.3%) of exon 21 were positive for L858R mutation in EGFR tyrosine kinase domain. Married persons and PDC were significant predictors of L858R mutation in EGFR tyrosine kinase domain in thyroid cancer as this was statistically significant in them with *p* = 0.04, 0.03 respectively.

**Conclusion:**

In our population, PTC is common in females with half of population harboring EGFR mutation and it is statistically significant in poorly differentiated carcinoma and in married individuals.

It implies that EGFR may be used in thyroid cancer as a possible therapeutic agent in our set of population.

**Supplementary Information:**

The online version contains supplementary material available at 10.1186/s43046-022-00139-y.

## Background

Thyroid cancer is one of the most common endocrine tumors worldwide [1, 2]. Papillary and follicular constitute about 90% among all types of thyroid malignancies [3, 4]. Prevalence of thyroid cancer is about 1–5% and 2% in females and males respectively. With the advent of new diagnostic modalities, the incidence of thyroid cancer is increasing [5]. There are various clinicopathologic criteria on which prognosis of thyroid cancer depends: age, gender, histologic subtype, tumor size, extrathyroidal extension (ETE), and the presence of lymph node (LNM) or distant metastases [6]. After a 20-year period of a relatively flat mortality rate, there has been a recent increase in thyroid carcinoma related mortality especially in men [7]. This has led to a greater interest in understanding tumor-specific markers in thyroid cancer to identify specific patients who can have adverse outcome.

Epidermal growth factor receptor (EGFR) refers to a mutation to the portion of DNA in a cancer cell (particularly lung cancer which carries the stimulus for making EGFR protein), allowing cancer cells to grow and spread. EGFR is one of the novel tumor markers, which has been studied extensively in lung cancer. EGFR is a transmembrane tyrosine kinase (TK) receptor. It is expressed in a variety of neoplastic and non-neoplastic tissues. Both mitogen activated protein kinase (MAPK) and phosphoinositide 3-kinase (PI3K) pathways are important for thyroid tumor progression. EGFR plays an important role in activating these pathways [8]. Papillary thyroid cancers (PTCs) expressing EGFR was demonstrated by Landriscina et al. and it is overexpressed by PTCs during dedifferentiation and anaplastic transformation [9]. EGFR overexpression is described in various thyroid malignancies: anaplastic thyroid cancers (ATCs), [10] follicular thyroid cancers (FTCs) [11, 12] and even medullary thyroid cancers (MTCs). However, some studies describe absence of somatic mutations in thyroid cancers [13, 14].

EGFR mutation is best characterized in lung adenocarcinoma, a specific activating mutation that affect the EGFR TK domain. The commonest mutations are deletions in exon 19 (del 2235-2249/2236-2250; del E746-A750), followed by a point mutation in exon 21 (T>G 2573) which results in substitution of leucine by arginine at codon 858 (L858R) [15]. Although similar somatic mutations are described in thyroid cancers [16] they are not well characterized [17]. However, there are some studies that fail to identify them [14, 18].

This study was carried to find out type of specific mutation that is incriminated in progression of thyroid cancer and their relationship with clinical parameters. Probably, it is first such kind of study in North India.

## Methods

A total of 60 (*n* = 60) histologically confirmed, previously untreated thyroid cancer patients attending Department of General and Minimal Access Surgery at a tertiary care center were included in this study. Tumor tissue along with corresponding normal tissue was available for all 60 patients. This was prospective cross-sectional study spanned for 2 years from January 2017 to January 2019. A written pre-informed consent was obtained from all cases. Demographic and clinicopathological characteristics of each patient were recorded in a questionnaire. In preoperative assessment of thyroid swelling, all those patients who had significant neck lymph node on Ultra sonography, were subjected to FNAC if it showed metastasis, neck dissection was carried out and on histopathology staging was done. Various clinical features were taken to find out relationship between thyroid cancer and EGFR mutation. Age is one of the prognostic factors in thyroid cancer, so we divided our patients into two groups: one with age > 45 years and other with age < 45 years and use of oral contraceptives is one of the risk factors associated with thyroid cancer. Somatic mutation screening was done on surgically resected and histopathological confirmed tumor and the corresponding normal tissues of thyroid cancer patients. EGFR mutational analysis was seen on exon 19, 20, 21. This study was approved by the Institutional Ethical committee.

### Sample collection/storage

The surgically resected tissue samples either by total thyroidectomy/hemi-thyroidectomy or lobectomy, were collected directly into sterile vials containing chilled PBS (Phosphate buffered saline) (pH = 7.2) and frozen at – 80 °C for molecular investigations. Adjacent normal tissues were resected from outside the margins of resection. Histopathologically, confirmed thyroid cancer tissues and corresponding normal tissues were used for mutational analysis of EGFR gene.

### Method used for extraction of DNA

DNA was extracted from the tissues by phenol-chloroform method and by Qiagen DNA extraction kit while salting out method was used for the extraction of DNA from blood samples.

The concentration of the DNA obtained was measured in a spectrophotometer at 260 nm wavelength by using the formula:

$$\mathrm{DNA}\ \upmu \mathrm{g}/\mathrm{ml}={A}_{260}\times 50\times \mathrm{dilution}\ \mathrm{factor}$$

The purity of DNA was checked by using *A*_260_/*A*_280_ ratio. The quality of the DNA obtained from the tissue specimens and blood samples was analyzed on 1% agarose gel. The high-molecular-weight DNA was used for further molecular investigation.

### Allele-specific PCR (AS-PCR)

**Fig. 1:**
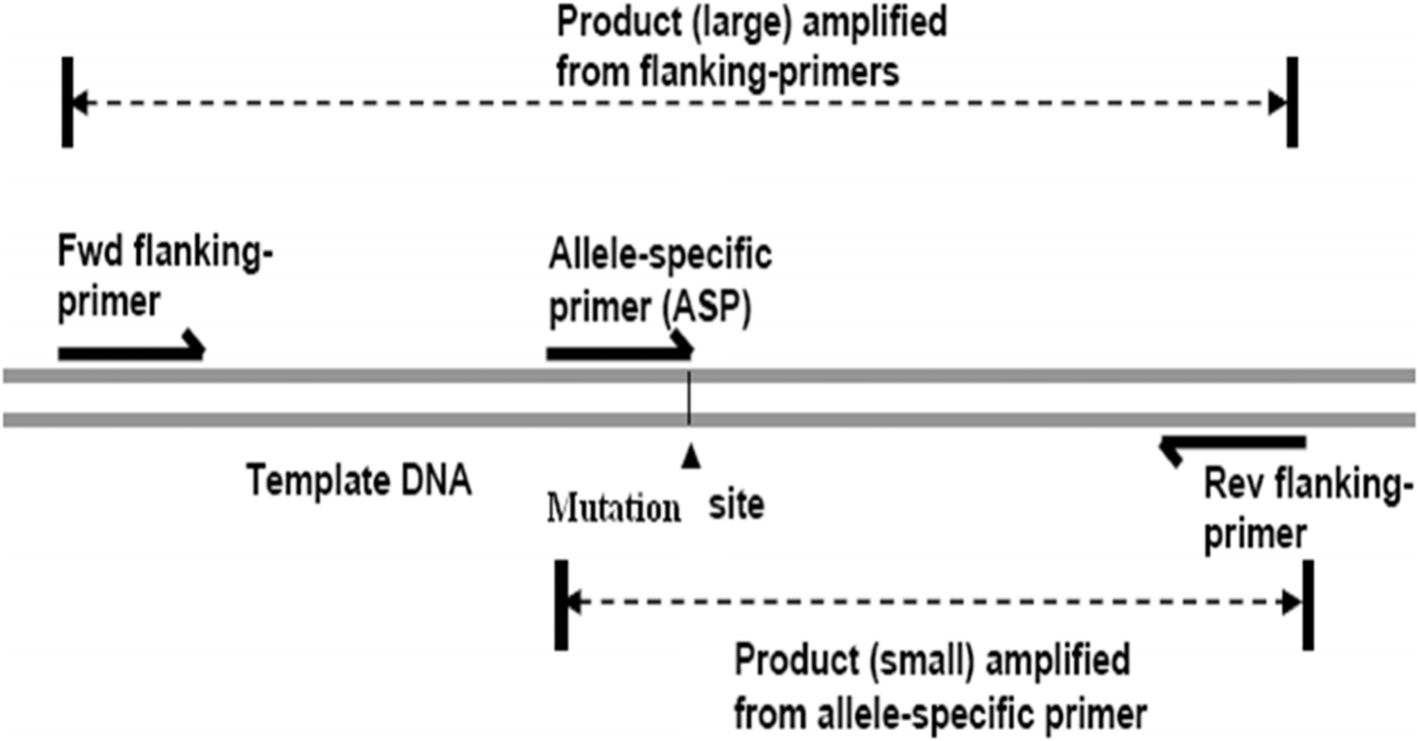
Schematic representation of AS-PCR

Given the high frequency of EGFR mutations and the possible implication of this receptor in the development of thyroid cancer, it was important to develop a simple, fast, and reliable method to identify these mutations in greater detail as a potential tool for the diagnosis and follow-up of these patients. The mutations in exon 19, 20, and 21 of EGFR gene account for more than 95% of total mutations in the gene. These mutations therefore represent an excellent target for assays, such as AS-PCR that depends on the specific detection of point mutations. The general principle underlying the AS-PCR technique is to design a mutation-specific primer that produces the preferential amplification of a specific mutant allele. The schematic representation of this AS-PCR is shown in Fig. 1.

**Fig. 2:**
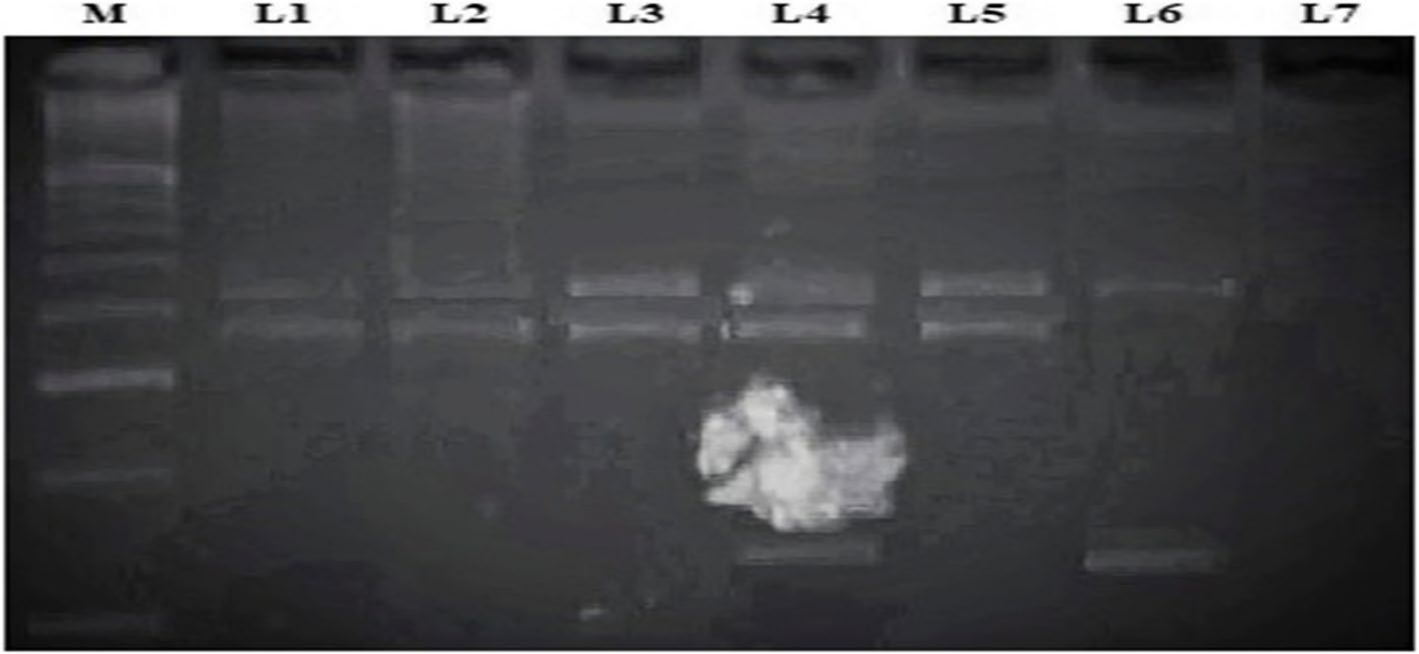
Representative picture of ARMS-PCR products for detection of 15 bp deletion in exon 19 of EGFR gene*. A* single tube reaction in which “M” contains molecular marker (100 bp); L1, L2, L3, and L4 contain 444 bp and 325 bp bands representing absence of deletion; L6 contains 444 bp and 134 bp bands representing the presence of 15 bp deletion; L4 and L6 contain 444 bp, 325 bp, and 134 bp bands representing the heterozygosity; L7 represents negative control

**Fig. 3:**
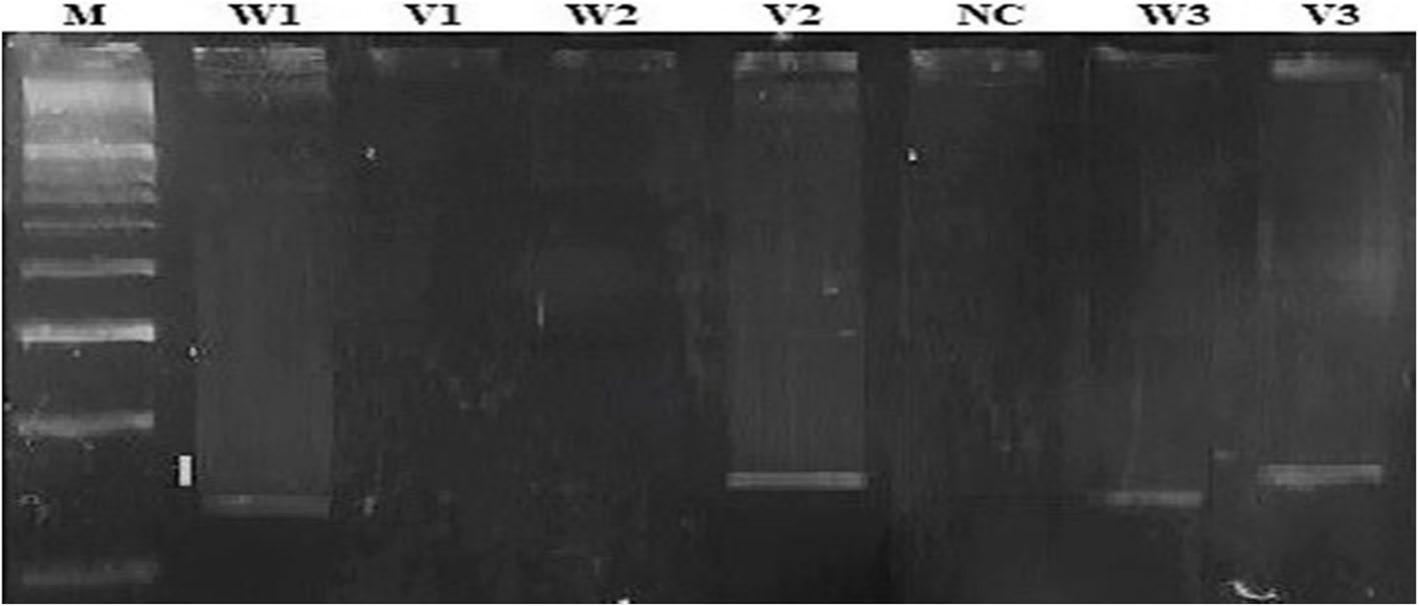
Representative picture of AS-PCR products for detection of T790M mutation in exon 20 of EGFR gene. Two tube reaction in which lanes marked as “W” contain bands pertaining to wild allele and “V” contains bands pertaining to variant allele of same sample. “M” contains molecular marker (100 bp); “W1” contains 139 bp band pertaining to wild type allele, “V1” contains 146 bp band pertaining to mutant/variant allele; “NC” represents negative control

**Fig. 4:**
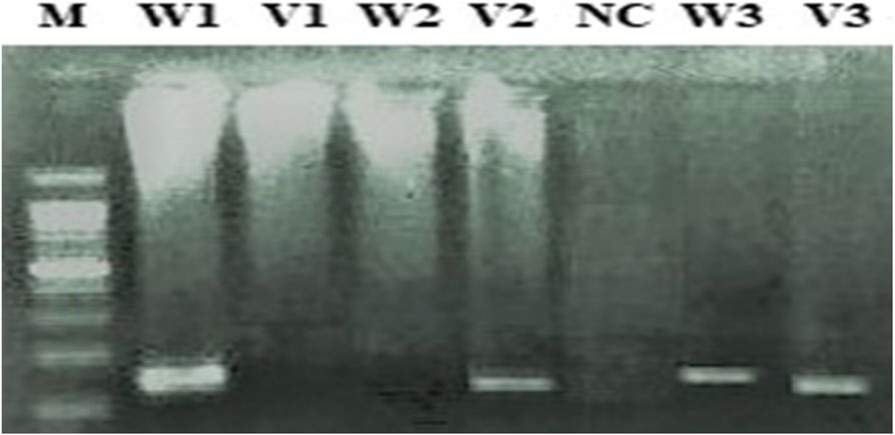
Representative picture of AS-PCR products for detection of L858R mutation in exon 21 of EGFR gene. Two tube reaction in which lanes marked as “W” contain bands pertaining to wild allele and “V” contains bands pertaining to variant allele of same sample. “M” contains molecular marker (100 bp); “W1” contains 137 bp band pertaining to wild type allele, “V1” contains 134 bp band pertaining to mutant/variant allele; “NC” represents negative control

1. Four primers were used in a single tube to setup an ARMS PCR for exon 19 (15 bp deletion; codons 746–750) of EGFR gene (Fig. 2)
2. Four primers were used in a two-tube reaction for setting up of AS-PCR for the detection of mutation in exon 20 (T790M) (Fig. 3)
3. Two allele specific primers and a single common primer were used in two tubes to determine the exon 21 mutations (L858R) in EGFR gene of thyroid cancer patients (Fig. 4, Table 1)
  For primers 14–25 nucleotides in length:
  $${T}_m=\left[{2}^{{}^{\circ}}C\times \left(\mathrm{number}\ \mathrm{of}\ \mathrm{A}\ \mathrm{and}\ \mathrm{T}\ \mathrm{bases}\right)\right]+\left[{4}^{{}^{\circ}}C\times \left(\mathrm{number}\ \mathrm{of}\ \mathrm{G}\ \mathrm{and}\ \mathrm{C}\ \mathrm{bases}\right)\right]$$

### Statistical analysis

Statistical analysis was performed by using SPSS software (V. 20.0). Chi-square test or Fisher’s test whichever appropriate for homogeneity of proportions was used to determine significance of mutation pattern and odds ratio was used to determine association of presence of mutations with various clinico-epidemiological characteristics such as age, site of tumor, clinical tumor stage, and histo-pathological grade of tumor. Statistical significance was considered when *p* < 0.05.

**Table 1:** Primers, product size and annealing temperatures used to detect mutations, if any, in various exons of *EGFR* gene by ARMS-PCR and AS-PCR

Amplicon	Change	Primer sequence	Annealing Temp. (°C)	Product size (bp)
Exon 19	15 bp deletion; codons 746–750	P-5′-GTAACATCCACCCAGATCACTG-3′ Q-5′-GTGTCAAGAAACTAGTGCTGGG-3′ A-5′-CCCGTCGCTATCAAGGAATTAA-3′ B-5′-GTTGGCTTTCGGAGATGTTTTGATAG-3′	60	(Single tube reaction) PQ = 444 bp (control) AQ = 325 bp (deletion absent) PB = 134 bp (deletion present)
Exon 20	T790M	E-5′-GAAGCCACACTGACGTGCCT-3′ F-5′-GCCGAAGGGCATGAGCTGTG-3′ G-5′-ACCATGCGAAGCCACACTGACG-3′ H-5′-GCCGAAGGGCATGAGCTGGA-3′	56	(Two tube reaction) EF = 139 bp (for wild allele) GH = 146 bp (for variant allele)
Exon 21	L858R (T2573G)	P-5′-GGGTCTTCTCTGTTTCAGGGCAT-3′ A-5′-TTCCGCACCCAGCAGTTTGGCTA-3′ B-5′-CGCACCCAGCAGTTTGGTTC-3′	60	(Two tube reaction) PA = 137 bp (wild allele) PB = 134 bp (variant allele)

## Results

As shown in Table 2 most of our patients were females 44 (73%). Forty-five (75%) of our population were in age group of < 45 years. Most of our patients were non-smokers 50 (83.3%). Neck swelling was the commonest presentation 55(91.6%). PTC 48(80%) was the commonest type of thyroid cancer. Lymph node metastasis was present in 32(53.4%) of patients.

**Table 2:** Demographic and clinico-pathological variables in our study population

Variable	Parameter	Cases (***n*** = 60)	
		***n***	%
**Gender**	Female	44	73.0
	Male	16	27.0
**Age in years**	< 45	45	75.0
	≥ 45	15	25.0
**Habitation**	Rural	51	85.0
	Urban	09	15.0
**Marital status**	Unmarried	25	41.6
	Married	35	53.4
**Use of OCP**^a^	Yes	05	8.4
	No	55	91.6
**Smoking status**	Non-smoker	50	83.3
	Smoker	10	16.7
**TSH levels** ^b^	Elevated	27	45.0
	Normal	33	55.0
**Serum Calcium**	Normal	40	66.6
	Decreased	20	33.4
**Initial PPX** ^c^	Swelling	55	91.6
	Incidental detection	05	8.4
**BTD** ^d^	Yes	05	8.4
	No	55	91.6
**Histological types**	Papillary	48	80.0
	Follicular	12	20.0
**Grade**	WD ^e^	41	68.4
	PD ^f^	19	31.6
**Stage, < 45 years**	Stage I	28	46.6
	Stage II	17	28.4
**Stage, ≥ 45 years**	Stages I and II	09	15.0
	Stage III/above	06	10.0
**LN**^a^ **metastasis**	Present	32	53.4
	Absent	28	46.6
**V/C** ^b^ **Invasion**	Present	17	28.4
	Absent	43	71.6

^a^Oral contraceptive

^b^Thyroid stimulating hormone

^c^PPX presentation

^d^Benign thyroid disease

^e^Well differentiated

^f^Poorly differentiated

^a^Lymph node

^b^Vascular capsular

EGFR 15 bp deletion (exon 19) was present in 32(53.3%) of patients, out of which 22 (68.75%) were females. There was more frequent involvement of this mutation in patients with high TSH level (> 5.5 IU/ml) which was statistically significant (*p* < 0.001). There was preponderance of PTC (29 vs 3) in patients bearing these mutations which was significant (*p* = 0.04) (Table 3).

**Table 3:** Association of EGFR 15 bp deletion in exon 19 with different variables of thyroid cancer patients

Variable	Cases ***n*** = 60	***EGFR*** ***15 bp deletion in exon 19*** (***n*** = 60)		OR (95% CI)	***P*** value
		Positive ***n*** = 32 (53.3%)	Negative ***n*** = 28 (46.7%)		
**Gender**				1.6(0.5–5.3)	0.39
**Female**	44(73.0)	22(50.0)	22(50.0)		
**Male**	16(27.0)	10(62.5)	06(37.5)		
**Age in years**				0.7(0.2–2.2)	0.55
**< 45**	45(75.0)	25(55.5)	20(44.4)		
**≥ 45**	15(25.0)	07(46.6)	08(53.4)		
**Habitation**				3.6(0.6–19.2)	0.15
**Rural**	51(85.0)	25(78.1)	26(50.9)		
**Urban**	09(15.0)	07(21.8)	02(22.2)		
**Marital status**				1.9(0.6–5.9)	0.02*
**Unmarried**	25(41.6)	09(36.0)	16(64.0)		
**Married**	35(53.4)	23(65.7)	12(34.3)		
**Use of OCP**				0.7(0.1–4.8)	0.25
**Yes**	05(8.4)	03(60.0)	02(40.0)		
**No**	55(91.6)	29(52.7)	26(47.3)		
**Smoking status**				4.3(0.8–22.4)	0.08
**Non-smoker**	50(83.4)	24(48.0)	26(52.0)		
**Smoker**	10(16.6)	08(80.0)	02(20.0)		
**TSH levels**				0.1(0.04–0.45)	< 0.001*
**Elevated**	27(45.0)	21(77.7)	06(22.3)		
**Normal**	33(55.0)	11(33.3)	22(66.7)		
**Serum calcium**				1.5(0.5–4.4)	0.46
**Normal**	40(66.6)	20(50.0)	20(50.0)		
**Decreased**	20(33.4)	12(60.0)	08(40.0)		
**Initial PPX**				0.5(0.08–3.5)	0.65
**Swelling**	55(91.6)	30(54.5)	25(45.4)		
**Incidental detection**	05(8.4)	02(40.0)	03(60.0)		
**BTD**				0.7(0.1–4.8)	0.25
**Yes**	05(80.0)	03(60.0)	02(40.0)		
**No**	55(20.0)	29(52.7)	26(47.3)		
**Histological types**				0.2(0.05–.09)	0.04*
**Papillary**	48(80.0)	29(60.5)	19(39.5)		
**Follicular**	12(20.0)	3(25.0)	09(75.0)		
**Grade**				2.5(0.7–7.8)	0.11
**WD**	41(68.4)	19(46.3)	22(53.7)		
**PD**	19(31.6)	13(68.4)	06(31.6)		
**Stage, < 45 years**				1.8(0.5–6.3)	0.33
**Stage I**	28(46.6)	14(50.0)	14(50.0)		
**Stage II**	17(28.4)	11(64.7)	06(35.3)		
**Stage, ≥ 45 years**				0.4(0.04–3.4)	0.60
**Stage I and II**	09(15.0)	05(55.5)	04(44.5)		
**Stage III and above**	06(10.0)	02(33.3)	04(66.7)		
**V/C invasion**				1.3(0.5–3.6)	0.58
**Present**	32(53.4)	16(50.0)	16(50.0)		
**Absent**	28(46.6)	16(57.1)	12(42.9)		
**LN metastasis**				2.8(0.9–9.0)	0.07
**Present**	17(28.4)	06(35.2)	11(64.8)		
**Absent**	43(71.6)	26(60.4)	17(39.6)		

*OCP* oral contraceptive, *TSH* thyroid stimulating hormone, *PPX* presentation, *BTD* benign thyroid disease, *WD* well differentiated, *PD* poorly differentiated, *VC* vascular capsular, *LN* lymph node

*P* value by chi-square test or Fisher’s exact test, whichever appropriate

*Statistically significant (*P* value < 0.05)

EGFR T790M (exon 20) was found in 5 (8.4%) patients. It was exclusively present in those tumors in which there was vascular/capsular invasion and it was statistically significant (*p* = 0.01) (Table 4).

**Table 4:** Association of EGFR T790M mutation in exon 20 with different variables of thyroid cancer patients

Variable	Cases ***n*** = 60	***EGFR*** T790M mutation (***n*** = 60)		OR (95% CI)	***P*** value
		Mutants ***n*** = 05 (8.4%)	Wild type ***n*** = 55 (91.6%)		
**Gender**				0.4(0.04–3.5)	0.31
**Female**	44(73.0)	05(11.4)	39(88.6)		
**Male**	16(27.0)	00(0.0)	16(100.0)		
**Age in years**				2.12(0.3–14.2)	0.59
**< 45**	45(75.0)	03(6.7)	42(93.3)		
**≥ 45**	15(25.0)	02(13.3)	13(86.6)		
**Habitation**				0.8(0.08–7.2)	1.00
**Rural**	51(85.0)	05(9.9)	46(90.1)		
**Urban**	09(15.0)	00(0.0)	09(100.0)		
**Marital status**				0.15(0.01–1.4)	0.15
**Unmarried**	25(41.6)	04(16.0)	21(84.0)		
**Married**	35(53.4)	01(1.9)	34(97.1)		
**Use of OCP**				0.7(0.07–6.8)	1.00
**Yes**	05(8.4)	00(0.0)	05(100.0)		
**No**	55(91.6)	05(9.0)	50(91.0)		
**Smoking status**				0.7(0.07–6.3)	0.57
**Non-smoker**	50(83.4)	05(10.0)	45(90.0)		
**Smoker**	10(16.6)	00(0.0)	10(100.0)		
**TSH levels**				3.5(0.3–34.1)	0.36
**Elevated**	27(45.0)	01(3.8)	26(96.2)		
**Normal**	33(55.0)	04(12.2)	29(87.8)		
**Serum calcium**				0.5(0.04–4.5)	0.65
**Normal**	40(66.6)	04(10.0)	36(90.0)		
**Decreased**	20(33.4)	01(5.0)	19(95.0)		
**Initial PPX**				1.4(0.1–13.8)	1.00
**Swelling**	55(91.6)	05(9.0)	50(91.0)		
**Incidental detection**	05(8.4)	00(0.0)	05(100.0)		
**BTD**				0.7(0.07–6.8)	1.00
**Yes**	05(80.0)	00(0.0)	05(100.0)		
**No**	55(20.0)	05(9.0)	50(91.0)		
**Histological types**				1.0(0.1–9.8)	1.00
**Papillary**	48(80.0)	04(8.3)	44(91.7)		
**Follicular**	12(20.0)	01(8.3)	11(91.7)		
**Grade**				0.5(0.05–4.9)	1.00
**WD**	41(68.4)	04(9.7)	37(90.3)		
**PD**	19(31.6)	01(5.3)	18(94.7)		
**Stage, < 45 years**				0.4(0.03–3.5)	0.27
**Stage I**	28(46.6)	03(10.7)	25(89.3)		
**Stage II**	17(28.4)	00(0.0)	17(100.0)		
**Stage, ≥ 45 years**				0.4(0.03–4.5)	0.48
**Stage I and II**	09(15.0)	02(22.3)	07(77.7)		
**Stage III and above**	06(10.0)	00(0.0)	06(100.0)		
**V/C invasion**				8.2(0.9–73.0)	0.01*
**Present**	32(53.4)	00(0.0)	32(100.0)		
**Absent**	28(46.6)	05(17.8)	23(82.2)		
**LN metastasis**				1.6(0.17–15.8)	1.00
**Present**	17(28.4)	01(5.8)	16(94.2)		
**Absent**	43(71.6)	04(9.4)	39(90.6)		

*OCP* = oral contraceptive, *TSH* = thyroid-stimulating hormone, *PPX* = presentation, *BTD* = benign thyroid disease, *WD* = well differentiated, *PD* = poorly differentiated, *VC* = vascular capsular, *LN* = lymph node

*P* value by chi-square test or Fisher’s exact test, whichever appropriate

*Statistically significant (*P* value < 0.05)

Prevalence of EGFR L858R mutation in our study population was 43.3% (26). 54.3% (19) married individuals were positive for this mutation and it was significant (*p* = 0.04). Twelve (63.25%) patients with poorly differentiated thyroid cancer bore this mutation and was statistically significant (*p* = 0.03) (Table 5).

**Table 5:** Association of EGFR L858R mutation in exon 21 with different variables of thyroid cancer patients

Variable	Cases ***n*** = 60	***EGFR L858R mutation*** (***n*** = 60)		OR (95% CI)	***P*** value
		Mutants ***n*** = 26 (43.3%)	Wild type ***n*** = 34 (56.7%)		
**Gender**				1.4(0.4–4.5)	0.52
**Female**	44(73.0)	18(41.0)	26(59.0)		
**Male**	16(27.0)	08(50.0)	08(50.0)		
**Age in years**				0.5(0.16–1.9)	0.36
**< 45**	45(75.0)	21(46.6)	24(53.4)		
**≥ 45**	15(25.0)	05(33.4)	10(66.6)		
**Habitation**				0.3(0.06–1.7)	0.27
**Rural**	51(85.0)	24(47.0)	27(53.0)		
**Urban**	09(15.0)	02(22.3)	07(77.7)		
**Marital status**				3.0(1.1–9.1)	0.04*
**Unmarried**	25(41.6)	07(28.0)	18(72.0)		
**Married**	35(53.4)	19(54.3)	16(45.7)		
**Use of OCP**				0.5(0.07–3.1)	0.64
**Yes**	05(8.4)	03(60.0)	02(40.0)		
**No**	55(91.6)	23(41.8)	32(58.2)		
**Smoking status**				0.8(0.2–3.3)	1.00
**Non-smoker**	50(83.4)	22(44.0)	28(56.0)		
**Smoker**	10(16.6)	04(40.0)	06(60.0)		
**TSH levels**				0.7(0.25–1.9)	0.49
**Elevated**	27(45.0)	13(48.1)	14(51.9)		
**Normal**	33(55.0)	13(39.3)	20(60.7)		
**Serum calcium**				1.5(0.5–4.4)	0.46
**Normal**	40(66.6)	16(40.0)	24(60.0)		
**Decreased**	20(33.4)	10(50.0)	10(50.0)		
**Initial PPX**				0.9(0.1–5.5)	1.00
**Swelling**	55(91.6)	24(43.6)	31(56.4)		
**Incidental detection**	05(8.4)	02(40.0)	03(60.0)		
**BTD**				3.3(0.3–31.7)	0.37
**Yes**	05(80.0)	01(20.0)	04(80.0)		
**No**	55(20.0)	25(45.5)	30(54.5)		
**Histological types**				0.9(0.25–3.3)	0.89
**Papillary**	48(80.0)	21(43.7)	27(56.3)		
**Follicular**	12(20.0)	05(41.6)	07(58.4)		
**Grade**				3.3(1.0–10.2)	0.03*
**WD**	41(68.4)	14(34.2)	27(65.8)		
**PD**	19(31.6)	12(63.2)	07(36.8)		
**Stage, < 45 years**				2.3(0.7–7.3)	0.007*
**Stage I**	28(46.6)	12(42.8)	16(57.2)		
**Stage II**	17(28.4)	14(82.3)	08(17.6)		
**Stage, ≥ 45years**				1.0(0.1–8.9)	1.00
**Stages I and II**	09(15.0)	03(33.4)	06(66.6)		
**Stage III and above**	06(10.0)	02(33.4)	04(66.6)		
**V/C invasion**				0.9(0.3–2.6)	0.94
**Present**	32(53.4)	14(43.7)	18(56.3)		
**Absent**	28(46.6)	12(42.8)	16(57.2)		
**LN metastasis**				2.3(0.7–7.6)	0.17
**Present**	17(28.4)	05(29.5)	12(70.5)		
**Absent**	43(71.6)	21(48.8)	22(51.2)		

*OCP* oral contraceptive, *TSH* thyroid-stimulating hormone, *PPX* presentation, *BTD* benign thyroid disease, *WD* well differentiated, *PD* poorly differentiated, *VC* vascular capsular, *LN* lymph node

*P* value by chi-square test or Fisher’s exact test, whichever appropriate

*Statistically significant (*P* value < 0.05)

## Discussion

Previous studies have failed to identify EGFR activating mutation in thyroid cancer [9, 19] in contradiction to our study. Possible reason could be that our study was focused on a specific population, i.e., Asian patients who were mostly non-smokers. As for NSCLC (non-small cell lung carcinoma) the EGFR-activating mutations are observed in a particular subset of patients; specifically, mutations are more common in never-smokers, women, Asians, and patients with adenocarcinoma. Lack of smoking history, the most common carcinogen of NSCLC, implies the possibility that other genetic and environmental factors contribute to the development of EGFR mutations.

Our study constituted predominantly females (73%) as compared to males (27%) is almost consistent with previous study [20] in which females are 2.9 times higher. Female preponderance could be due to polymorphism role of estrogen receptor [9, 19, 21]. There is a significant cell proliferation in thyroid cancer tissue in females due to estrogen [21]. PTC was the commonest histologic type in our study population, which is consistent with other study [22].

High TSH (> 5.5 IU/ml) was predominately found in patients with EGFR 15 bp deletion and it was significant. Although there are no such studies in literature that have found such relationship, but hypothyroid status has been found to be linked to thyroid malignancy [23, 24].

The overall 15 bp deletion rate in EGFR exon 19 among 60 patients was found to be 53.3% (32/60). Out of them, 60.5% (29/48) deletions were detected in PTC and only 25.0% (03/12) in FTC. Exon 19 mutational study was also done in relation to lung cancers and it was found that the EGFR exon 19 insertions are a newly appreciated family of EGFR-TKI–sensitizing mutations, and patients with tumors harboring these mutations should be treated with EGFR-TKI. While these mutations may be missed through the use of some mutation-specific assays, the addition of PCR product size analysis to multi-gene assays allows sensitive detection of both exon 19 insertion and deletion mutations [25]. Nevertheless, detailed studies were not done in case of thyroid malignancy. In the future, we can predict the use of tyrosine kinase inhibitors [TKIs] as a treatment modality for advanced/undifferentiated thyroid malignancy in this part of world.

Many studies showed the presence of T790M gene mutations with EGFR domain of exon 20, these studies were conducted in lung carcinoma and the effect of targeted based TKIs was studied in detail [26, 27]. The total mutational rate of T790M in EGFR tyrosine kinase domain (exon 20) among 60 patients was found to be only 8.4% (05 of 60). Only 8.3% of mutations were detected in PTC (04 of 48) as well as in FTC (01 of 12) patients (*P* > 0.05). Importance of T790M gene mutations with thyroid malignancy needs a detailed study.

The total of 43.3% (26 of 60) of thyroid cancer patients were positive for L858R mutation in EGFR tyrosine kinase domain of exon-21. Though probably such mutation has not been studied in thyroid cancer, there are studies in which similar type has been studied in lung cancer [28, 29]. There was preponderance of L858R mutation in our married cohort, which was statistically significant. SEER study [30] which was conducted between 2002 and 2007 to find out effect of marital status and various other factors on the prognosis of cancer. It showed married people had better cancer specific survival than unmarried (*p* < 0.05) ones. The presence of these mutations in married individuals with thyroid cancer needs elaborative study.

## Conclusion

In our study, we extensively studied the role of EGFR mutations with thyroid cancers, and various mutations in exon 19, 20, and 21.Significant relations of multiple variables were seen in associations with above described EGFR domain. This study will provide a nidus for future scope of further elaborating the clinical aspect of EGFR in the management of differentiated as well as aggressively behaving anaplastic thyroid malignancy in our part of world and at the same time will encourage many to take this study further in assuming a vital background for more elaborate work on thyroid malignancy.

## Supplementary Information

**Additional file 1.**

## Data Availability

It is not publicly available but is available with the corresponding author.

## Abbreviations

EGFR: Epidermal growth factor receptor
PTC: Papillary thyroid carcinoma
PDC: Poorly differentiated carcinoma
WDC: Well-differentiated carcinoma
FTC: Follicular thyroid carcinoma
TKI: Tyrosine kinase inhibitor
NSCLC: Non-small cell lung carcinoma
PCR: Polymerase chain reaction
AS-PCR: Allele specific polymerase chain reaction
MTC: Medullary thyroid carcinoma
MAPK: Mitogen activated protein kinase
TSH: Thyroid-stimulating hormone
PI3K: Phosphoinositide 3-kinase
SEER: Cancer statistics, trends, and multiple primary cancer analyses from the surveillance, epidemiology, and end results
ARMS: Amplification refractory mutation system
ATC: Anaplastic thyroid cancer
PBS: Phosphate-buffered saline
LNM: Lymph node metastasis
ETE: Extrathyroidal extension
