# Supplementary material for: Spectrum of EGFR mutation and its relation with high-risk predictors in thyroid cancer in Kashmiri population: 2 years prospective study at a tertiary care hospital
Source: J Egypt Natl Canc Inst. 2022 Oct 17;34:43. doi: 10.1186/s43046-022-00139-y (PMC13314227; doi:10.1186/s43046-022-00139-y)
Supplement: Supplementary file 1 — Additional file 1. [file 43046_2022_139_MOESM1_ESM.docx]

**4.1 Chemicals and Reagents**

The various chemicals and molecular biology reagents utilized during my research were procured from standard sources.

**4.2 Specimen**

*Patients and Controls*

A total of sixty (n=60) histologically confirmed, previously untreated Thyroid cancer patients attending Department of General and Minimal Invasive Surgery, Sher-I-Kashmir Institute of Medical Sciences (SKIMS), Srinagar were included in this study. Tumour tissue along with corresponding normal tissue was available for all 60 patients. A written pre informed consent was obtained from all cases and controls. Demographic and clinicopathological characteristics of each patient were recorded in a Questionnaire. This study was approved by the Ethical committee of the SKIMS.

*Sample collection/storage*

The surgically resected tissue samples either by total thyroidectomy/hemi-thyroidectomy or Lobectomy, were collected directly into sterile vials containing chilled PBS( Phosphate buffered saline) (pH=7.2) and frozen at -80°C for molecular investigations. Adjacent normal tissues were resected from outside the margins of resection. Histopathologically confirmed Thyroid cancer tissues and corresponding normal tissues were used for mutational analysis of *EGFR* gene.

**4.3 Extraction and Quantitation of genomic DNA**

*Principle*

DNA is extracted from the mammalian cells by lysing the cell membranes using detergents like SDS. The protein content of the cells is then precipitated either by organic solvents like phenol-chloroform-isoamyl mixture or by various salts such as sodium chloride, ammonium acetate or potassium acetate. Finally, the DNA is precipitated by ethanol or isopropanol and the DNA pellet is dissolved in Tris- EDTA.

DNA was extracted from the tissues by Phenol - Chloroform method and by Qiagen DNA extraction kit while Salting out method was used for the extraction of DNA from blood samples.

*Methodology of DNA extraction*

High-molecular-weight DNA was isolated by using *proteinase-K* and phenol method

1. The frozen tissue was allowed to thaw at room temperature.
2. The tissue was chopped with fresh surgical blades in a sterile petri dish.
3. The chopped tissue was then transferred into a sterile polypropylene tube (15ml) containing 3ml of 1X TE (see appendix II), 2ml of lysis buffer (see appendix II) and *Proteinase-K* to a final concentration of 100μg/ml was added to it.
4. The mixture was incubated at 37°C in a water bath for overnight.
5. Next day, equal volume of TE saturated phenol (see appendix II) was added and the mixture was gently mixed by inversion of tubes on overhead shaker for 15 minutes.
6. The tubes were then centrifuged at 3000-4000 rpm at 4°C for 15 minutes.
7. The supernatant aqueous phase was collected in a fresh polypropylene tube without disturbing the interphase with the help of a micropipette fitted with a wide bored tip.
8. To the supernatant from above step, equal volume of TE saturated phenol chloroform-isoamyl alcohol (25:24:1) was added and the mixture was shaken on overhead shaker for 15 minutes and steps 6 and 7 repeated.
9. To the supernatant thus obtained in fresh tube, equal volume of chloroform isoamyl alcohol (24:1) was added and each tube was shaken, and step 6 and 7 repeated.
10. To the supernatant from the above step, 1/10 volume of chilled 3M sodium acetate solution (pH=5.2) and 2.5 volumes of chilled ethanol or equal volume of isopropanol was added and mixed by gently inverting the tube. If visible precipitate of genomic DNA appeared, it was transferred to 1.5ml microfuge tube and centrifuged at 6000 rpm for 5 minutes. The pellet thus obtained was washed with 500μl of 70% ethanol and re-centrifuged. The washing was repeated.
11. If the precipitate of genomic DNA was not visible, the tubes were then allowed to stand at either -70°C for 10 minutes or at -20°C for overnight. Next day, the tube was centrifuged at 6000 rpm at 4°C for 45 minutes. The pellet thus obtained was washed twice with 70% ethanol as above.
12. Air/vacuum dried DNA pellet was dissolved in 200μl of DNA storage buffer and stored at 4°C or at -20°C for longer periods.

*Quantitation*

The concentration of the DNA obtained was measured in a spectrophotometer at 260nm wavelength by using the formula:

DNA μg/ml = A_260_ x 50X dilution factor.

The purity of DNA was checked by using A_260_/ A_280_ ratio. The quality of the DNA obtained from the tissue specimens and blood samples was analyzed on 1% agarose gel. The high-molecular-weight DNA was used for further molecular investigation.

**4.4 Polymerase chain reaction**

*Principle and application*

Polymerase chain reaction (PCR), a powerful technique developed by a team headed by *Kary Mullis* at Cetus Corporation, is used to amplify a segment of DNA in vitro *.* This method can produce large amount of a specific DNA sequence from a complex DNA template in a simple enzymatic reaction. This method utilizes a DNA polymerase and two oligonucleotide primers to synthesize a specific DNA from a single stranded template sequence. The oligonucleotides typically have different sequences and are complementary to sequences that lie on opposite strands of the template DNA and flank the segment of DNA that is to be amplified. The length of the primers usually 20 bases or more must be sufficient to overcome the statistical likelihood that their sequence would occur randomly in the overwhelmingly large number of non-target DNA sequences in the sample. PCR is carried out in a series of cycles. Each cycle begins with a denaturation step to render the target DNA single stranded. This is followed by an annealing step during which the primers anneal to their complementary sequences so that their 3' hydroxyl ends face the target. Finally, each primer is extended through the target region by the action of DNA polymerase. Since the products of one round of amplification serve as templates for the next cycle, the three step cycles are repeated until a sufficient amount of the product is produced. The major product of this exponential reaction is a segment of double-stranded DNA whose termini are defined by the 5' termini of the oligonucleotide primers and whose length is defined by the distance between the primers. In addition, longer DNA molecules are generated during the reaction. For example, the products of a successful first round of amplification are heterogeneously sized DNA molecules, whose lengths may exceed the distance between the binding sites of the two primers. In the second round these molecules generate DNA molecules of defined length that will accumulate in an exponential fashion in the later rounds of amplification and will form the dominant products of the reaction. Although longer molecules continue to be formed from the original DNA template in every round, they accumulate only at a linear rate and therefore do not contribute significantly to the final product.

The earliest PCR experiments utilized the Klenow fragment of *Escherichia* *coli* DNA polymerase I at a temperature of 37°C and often produced incompletely pure target products as judged by gel electrophoresis. However, the isolation of a heat-resistant DNA polymerase from *Thermus* *aquaticus* (*Taq*) allows primer annealing and extension to be carried out at an elevated temperature, thereby reducing mismatched annealing to non-target sequences. This added selectivity results in the production of large amounts of virtually pure target DNA.

Another important advantage of *Taq* polymerase is that it escapes inactivation at higher temperatures and need not be replaced after every denaturation step. This has allowed automation of PCR using machines that have controlled heating and cooling capability. This results in substantial improvements in the specificity and yield of the amplification reactions and the size of the amplified product. For example, between 0.5μg and 1.0μg of target DNA up to 2kb in length can be obtained from 30-35 cycles of amplification with only 10-6μg of genomic DNA.

*Allele-specific PCR (AS-PCR)*

Given the high frequency of *EGFR* mutations and the possible implication of this receptor in the development of thyroid cancer, it was important to develop a simple, fast, and reliable method to identify these mutations in greater detail as a potential tool for the diagnosis and follow-up of these patients. The mutations in exon 19, 20 and 21 of EGFR gene account for more than 95% of total mutations in the gene. These mutations therefore represent an excellent target for assays, such as allele-specific PCR (AS-PCR) that depends on the specific detection of point mutations. The general principle underlying the AS-PCR technique is to design a mutation-specific primer that produces the preferential amplification of a specific mutant allele. The schematic representation of this AS-PCR is shown in *Figure 1..*


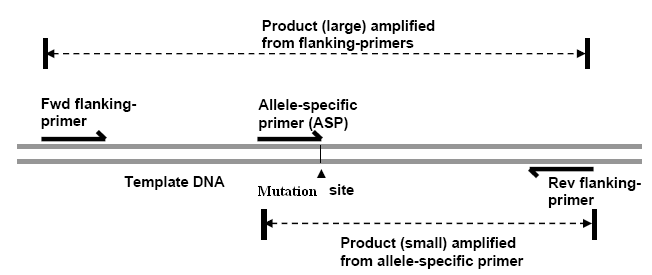


**Figure 4.1:** Schematic representation of Allele-specific PCR.

*Procedure for AS-PCR amplification of various exons of EGFR genes*

All precautions were taken to ensure contamination free amplification of DNA. Prevention of carry-over by separation of pre and post-PCR steps, and use of aerosol-free tips were strictly followed. Results were considered valid only on reproduction of same in two independent experiments.

*Equipment’s and Reagents*

- 1. Thermal cycler (Eppendorf Thermocycler).
  2. Micropipettes
  3. 0.2 ml PCR tubes
  4. Genomic DNA: 250 ng/μl
  5. 10X PCR buffer: 100mM Tris-HCL, pH 8.3; 500mM KCL; 15mM MgCl_2_; 0.1% gelatine and 1% Triton X100.
  6. Deoxyribonucleotide triphosphate: 10mM each dATP, dCTP, dGTP and dTTP.
  7. Primers: 10μM in sterile deionised water.
  8. *Taq* DNA polymerase: 5U/μl.

*Primers for amplification*

1. Four primers were used in a single tube to setup an ARMS PCR for exon 19 (15 bp deletion; codons 746-750) of EGFR gene (Table 3.1).
2. Four primers were used in a two tube reaction for setting up of AS-PCR for the detection of mutation in exon 20 (T790M) (Table 3.1).
3. Two allele specific primers and a single common primer were used in two tubes to determine the exon 21 mutations (L858R) in EGFR gene of thyroid cancer patients (Table 3.1).

*Protocol*

The amplification reaction was carried out in 25μl reaction volume in a 0.2ml PCR tubes. The reaction contained the following reagent volumes: -

1. 10X PCR buffer 2.5μl

2. 10mM dNTP mix 0.5μl

3. Primer no. 01 0.5μl

4. Primer no. 02 0.5μl

5. Primer no. 03 0.5μl

6. Primer no. 04 0.5μl

5. *Taq* DNA polymerase (5U/ μl) 0.2μl

6. Genomic DNA 1.0μl

7. Distilled water (25-rest of components) μl

*Total volume 25.0μl*

The above mentioned reagents were pipetted in a 0.2ml thin walled PCR tube and placed in Thermocycler. The following temperature profile was used for amplification: -

1. Initial denaturation 95°C for 4 minutes

2. Denaturation 95°C for 30 seconds

3. Annealing x°C for 30 seconds*

4. Extension 72°C for 30 seconds

5. Final Extension 72°C for 7 minutes

Temperature profile from step 2-4 was used for 35 cycles before final extension

*x was 3-5°C lower than the melting temperature (T_m_) of the primers and was calculated by using the following formulae.

1. For primers 14-25 nucleotides in length:

T_m_ = [2°C x (number of A and T bases)] + [4°C x (number of G and C bases)]

| Amplicon | Change | Primer sequence* | Annealing  Temp. (°C) | Product  size (bp) |
| --- | --- | --- | --- | --- |
| Exon 19 | 15 bp deletion; codons 746-750 | P - 5’-GTAACATCCACCCAGATCACTG-3’  Q - 5’-GTGTCAAGAAACTAGTGCTGGG-3’  A - 5’-CCCGTCGCTATCAAGGAATTAA-3’  B - 5’-GTTGGCTTTCGGAGATGTTTTGATAG-3’ | 60 | (Single tube reaction)  PQ=444bp (control)  AQ=325bp (deletion absent)  PB=134bp (deletion present) |
| Exon 20 | T790M | E - 5’-GAAGCCACACTGACGTGCCT-3’  F - 5’-GCCGAAGGGCATGAGCTGTG-3’  G - 5’-ACCATGCGAAGCCACACTGACG-3’  H - 5’-GCCGAAGGGCATGAGCTGGA-3’ | 56 | (Two tube reaction)  EF= 139bp (for wild allele)  GH=146bp (for variant allele) |
| Exon 21 | L858R  (T2573G) | P - 5’-GGGTCTTCTCTGTTTCAGGGCAT-3’  A - 5’-TTCCGCACCCAGCAGTTTGGCTA-3’  B - 5’-CGCACCCAGCAGTTTGGTTC-3’ | 60 | (Two tube reaction)  PA=137 bp (wild allele)  PB=134 bp (variant allele) |

**Table 1:** Primers, product size and annealing temperatures used to detect mutations, if any, in various exons of *EGFR* gene by ARMS-PCR and AS-PCR.

*Detection of PCR products*

The amplified products were detected and confirmed by comparing with a 100bp DNA marker ladder by electrophoresis on 3% agarose gel containing ethidium bromide (0.5μg/ml) in a mini gel system, as described in the section on agarose gel electrophoresis with few changes which are listed below: -

1. To 3g of agarose, 100ml of 1X TAE buffer was added in a conical flask and placed in boiling water bath until agarose was completely dissolved.

2. After placing the well set gel with buffer in the electrophoresis unit, 10μl of AS-PCR products mixed with 2μl of 6X gel loading buffer was loaded into the wells along with 100bp DNA marker ladder.

3. Electrophoresis was carried out at 50 volts until dye had migrated a sufficient distance through the gel. The electric current was turned off and the gel was examined on a UV illuminator and photographed.
